# Xenografted human microglia display diverse transcriptomic states in response to Alzheimer’s disease‐related Aβ pathology

**DOI:** 10.1002/alz.090394

**Published:** 2025-01-03

**Authors:** Renzo Mancuso

**Affiliations:** ^1^ Flanders Institute for Biotechnology, Antwerp Belgium

## Abstract

**Background:**

Microglia are central players in Alzheimer’s Disease (AD) pathology, but analyzing microglia states in human brain samples is challenging due to genetic diversity, postmortem delay and admixture of pathologies.

**Method:**

To circumvent these issues, here we collected 138,577 single cell expression profiles of human stem cell derived‐microglia from a xenotransplantation model of AD.

**Result:**

Xenografted human microglia adopt a disease‐associated (DAM) profile similar to that seen in mouse microglia, but display a more pronounced HLA state, likely related to antigen presentation in response to amyloid plaques. The human microglial response also involves a pro‐inflammatory cytokine/chemokine CRM response to oligomeric Aβo. Expression of AD risk genes is distributed over this multi‐pronged response to amyloid pathology. Genetic deletion of *TREM2* or *APOE*, as well as *APOE* polymorphisms and *TREM2^R47H^* expression in the transplanted microglia modulate these responses differentially.

**Conclusion:**

We have identified multiple transcriptomic cell states adopted by microglia in response to AD‐related pathology, which should be taken into account in translational studies.

